# Oriented graphene nanoribbons embedded in hexagonal boron nitride trenches

**DOI:** 10.1038/ncomms14703

**Published:** 2017-03-09

**Authors:** Lingxiu Chen, Li He, Hui Shan Wang, Haomin Wang, Shujie Tang, Chunxiao Cong, Hong Xie, Lei Li, Hui Xia, Tianxin Li, Tianru Wu, Daoli Zhang, Lianwen Deng, Ting Yu, Xiaoming Xie, Mianheng Jiang

**Affiliations:** 1 State Key Laboratory of Functional Materials for Informatics, Shanghai Institute of Microsystem and Information Technology, Chinese Academy of Sciences, 865 Changning Road, Shanghai 200050, China; 2 School of Physical Science and Technology, ShanghaiTech University, 319 Yueyang Road, Shanghai 200031, China; 3 School of Optical and Electronic Information, Huazhong University of Science and Technology, Wuhan 430074, China; 4 School of Physics and Electronics, Central South University, Changsha 410083, China; 5 Graduate University of the Chinese Academy of Sciences, Beijing 100049, China; 6 Division of Physics and Applied Physics, School of Physical and Mathematical Sciences, Nanyang Technological University, 21 Nanyang Link, Singapore 637371, Singapore; 7 State Key Laboratory of ASIC & System, School of Information Science and Technology, Fudan University, Shanghai 200433, China; 8 National Laboratory for Infrared Physics, Shanghai Institute of Technical Physics, Chinese Academy of Sciences, 500 Yu Tian Road, Shanghai 200083, China

## Abstract

Graphene nanoribbons (GNRs) are ultra-narrow strips of graphene that have the potential to be used in high-performance graphene-based semiconductor electronics. However, controlled growth of GNRs on dielectric substrates remains a challenge. Here, we report the successful growth of GNRs directly on hexagonal boron nitride substrates with smooth edges and controllable widths using chemical vapour deposition. The approach is based on a type of template growth that allows for the in-plane epitaxy of mono-layered GNRs in nano-trenches on hexagonal boron nitride with edges following a zigzag direction. The embedded GNR channels show excellent electronic properties, even at room temperature. Such in-plane hetero-integration of GNRs, which is compatible with integrated circuit processing, creates a gapped channel with a width of a few benzene rings, enabling the development of digital integrated circuitry based on GNRs.

## Abstract (web summary)

Graphene nanoribbons are promising candidates for 2D material electrical interconnects; however, the top-down fabrication of nanoribbons has remained a challenge. Here, Chen *et al*. have used a hexagonal boron nitride template to grow narrow, integrated graphene nanoribbons with small bandgaps.

Ideal graphene nanoribbons (GNRs) have been shown to exhibit extreme chirality dependence as metals or semiconductors^1^. Therefore, the capability to precisely produce GNRs with defined chirality at the atomic level is required in order to engineer their band gap and electrical properties^2^,^3^. Conventional lithography^4^,^5^,^6^ always results in ragged edges along the GNRs. Other GNR synthesis methods, including sidewall growth on SiC^7^, advanced lithography^8^, sono-chemical methods^9^,^10^ and carbon nanotube unzipping^11^,^12^, still present difficulties for either chirality control or width-scaling down to 10 nm and less. Recently, bottom-up synthesis methods using catalytic substrates^13^,^14^,^15^ were demonstrated to form GNRs with well-defined edge structures and atomic precision. However, transferring techniques to produce devices without degrading the quality of the GNRs remain a formidable challenge. It is obvious that earlier approaches have fundamental limitations for further electronic investigation. Electronics always require scalable transfer-free approaches for growing GNRs and conducting band gap engineering. Controlled fabrication of oriented GNRs embedded on hexagonal boron nitride (h-BN) has the capability to overcome the above difficulties. With proper control, the band gap and magnetic properties can be precisely engineered. Most desired features for GNRs can be automatically attained using this approach.

Here, we demonstrate the successful growth of GNRs directly on h-BN substrates with smooth edges and controllable widths via templated growth using chemical vapour deposition (CVD). Transistors made with sub-10-nm GNRs demonstrate large on–off ratios of more than 10^4^ at room temperature and carrier mobility values of ∼750 cm^2^ V^−1^ s^−1^. For the narrowest GNRs, the band gaps extracted from the electrical transport data are >0.4 eV.

## Results

### Templated growth of oriented GNRs in h-BN trenches

**Figure 1:**
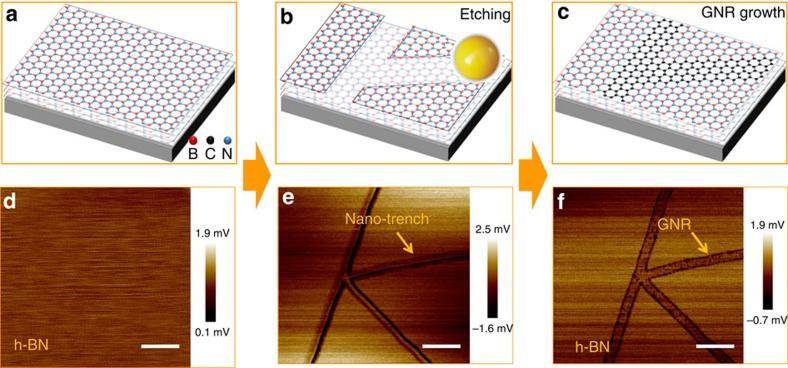
Formation of GNRs in h-BN trenches. (**a**) Smooth surface of the h-BN; (**b**) Synthesis of nano-trenches on h-BN by Ni particle-assisted etching; (**c**) In-plane epitaxial template growth of GNRs via CVD; (**d**–**f**) AFM friction images corresponding to the schematics shown in **a**–**c**, respectively. The friction images showed better contrast than the height images, especially for GNRs embedded in the h-BN nano-trenches. Scale bars, 200 nm.

Figure 1 demonstrates our conceptual design and its experimental verification for the synthesis of GNRs on h-BN via templated growth (details of the experimental procedures are given in the ‘Methods' section). Single-crystal h-BN flakes exhibited a very smooth surface after multiple cleaning steps for removing possible surface contaminants (see the atomic force microscopy (AFM) images in Fig. 1d and Supplementary Fig. 1a,b). The nano-trenches on h-BN were synthesized by nickel particle-assisted etching (Fig. 1b,e and Supplementary Fig. 1c,d). After CVD growth, the trenches were filled with graphene (Fig. 1c,f and Supplementary Fig. 1e,f).

### AFM measurement of oriented trenches in h-BN crystals

The nano-trenches exhibited predominant separation angles of 60° relative to each other with no obvious correlation to the direction of the gas flow, indicating anisotropic etching along the different crystallographic directions (Supplementary Figs 2 and 3). Separation angles of 30° and 90° were rarely observed. The lattice structures shown in the inset of Supplementary Figs 2a,b and 3a confirm that the crystallographic orientations of the h-BN trenches/edges follow a zigzag (ZZ) pattern. The crystallographically selective chemical reaction indicates lower activation energy along the ZZ patterns of the h-BN.

**Figure 2:**
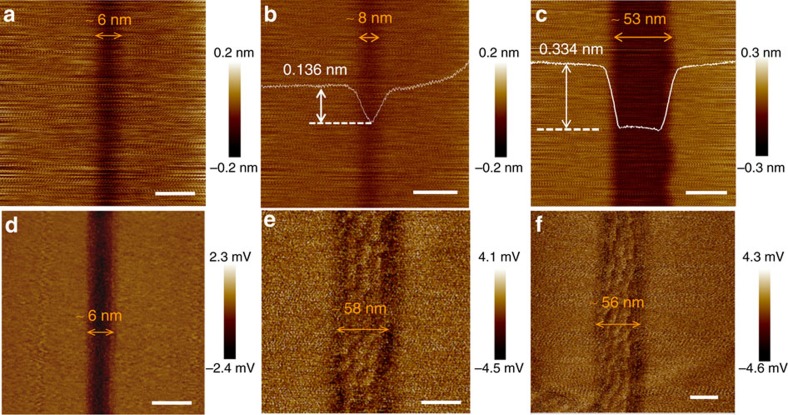
Images of nano-trenches and GNRs on the h-BN substrate. AFM height images of mono-layered nano-trenches on the h-BN crystal with the following widths: (**a**) ∼6 nm, scale bar: 10 nm, (**b**) ∼8 nm, scale bar: 20 nm and (**c**) ∼53 nm, scale bar: 40 nm. AFM friction images of graphene nanoribbons (GNRs) embedded in the nano-trenches on the h-BN crystal via template growth with the following widths: (**d**) ∼6 nm, scale bar: 10 nm, (**e**) ∼58 nm, scale bar: 40 nm and (**f**) ∼56 nm, scale bar: 40 nm. The giant Moiré pattern can be observed in **e**,**f**, indicating that the GNRs are precisely aligned with the h-BN.

Figure 2a–c shows some examples of nano-trenches obtained on h-BN substrates. The width of the trenches and their distribution were strongly dependent on the etching parameters and solutions (see Supplementary Table 1). Nanometre-sized-trenches, narrower than 10 nm in width, could be reproduced via an optimized process. Wider nano-trenches (Fig. 2c) could be synthesized by increasing the etching duration or temperature (see Supplementary Table 1). The trench in Fig. 2c had a depth of 0.334 nm with a bottom roughness comparable to that of pristine h-BN, indicating that the trench was mono-layered. The extremely anisotropic two-dimensional (2D) nature of h-BN predominantly confined the etching to a single-atom layer. Due to the size limitation of the AFM tips, the depth of the narrower trenches could not be exactly determined, but these trenches were reasonably judged to also be mono-layered based on the measurements of wider nano-trenches. The tip size limitation may also result in uncertainty in the width measurements of the nano-trenches.

### AFM measurement of oriented GNRs on h-BN crystals

Figure 2d–f presents typical AFM friction images of GNRs embedded in the nano-trenches of the h-BN substrate, and the corresponding height images are given in Supplementary Fig. 4. A super-lattice structure, also known as a Moiré pattern, is clearly visible in Fig. 2e,f for the synthesized GNRs with widths of ∼58 and ∼56 nm, respectively, which was better contrasted in the friction images than in the height images. For narrow GNRs, a Moiré pattern was not observed (Fig. 2d and Supplementary Fig. 4a) because the GNR width was comparable to or smaller than the periodicity of the Moiré pattern. Height variations in the range of 20–40 pm could be seen near the boundary of the graphene/h-BN, which is most likely due to lattice distortions caused by mismatched lattice constants and coefficients of thermal expansion between the graphene and h-BN. The small out-of-plane distortions also excluded the possibility of the formation of multilayered GNRs. More height images of the GNRs are shown in Supplementary Fig. 5.

The existence of the giant Moiré pattern indicates that the graphene was highly crystalline and precisely aligned with the h-BN underneath. It was noticed that the Moiré pattern appeared to be stretched along the GNR, while it appeared relaxed laterally. This trend differs from regular hexagons with a periodicity of ∼14 nm, which have always been observed with well-aligned graphene domains on h-BN^16^,^17^,^18^. This observation gives a strong indication of the in-plane epitaxy between the graphene and the h-BN at the edges of the trench, where the graphene is stretched by tensile strain along the ribbon, due to a lattice mismatch between the graphene and h-BN. Supplementary Fig. 6 illustrates how the stretched Moiré pattern formed, and Supplementary Fig. 7 shows the dependence of its wavelength along the stretched direction on the strain level. According to the relationship shown in Supplementary Fig. 7a, the strain in the GNR along the ribbon was estimated to be ∼0.75±0.3% from the wavelength of the stretched Moiré pattern measured in the AFM images. In addition, atomic-resolution AFM images confirmed that the in-plane connections between the GNRs and h-BN were continuous (see Supplementary Fig. 8).

Supplementary Fig. 9 shows AFM images taken before, during and after the GNR growth, illustrating that the graphene grew via a step-flow mechanism from two step-edges of the h-BN top-layer trench and coalesced into a complete GNR. The growth of the GNRs was also observed to occur at a one-sided atomic step-edge, which was formed by mechanical cleavage. Supplementary Fig. 10 shows a case in which the nanoribbon grew along the atomic step-edge of the top layer on the h-BN and developed laterally. Atomic-resolution images show that there were no discernible rotational misalignments between the three lattices (the graphene epi-ribbon, the top h-BN layer and the underlying h-BN lattice), indicating that while the GNR developed, the chirality of its edge was unchanged. Of course, in this case, the width of the GNR was determined by the growth time, not by the nano-trench confinement.

### Raman characterization of GNRs embedded in the h-BN

**Figure 3:**
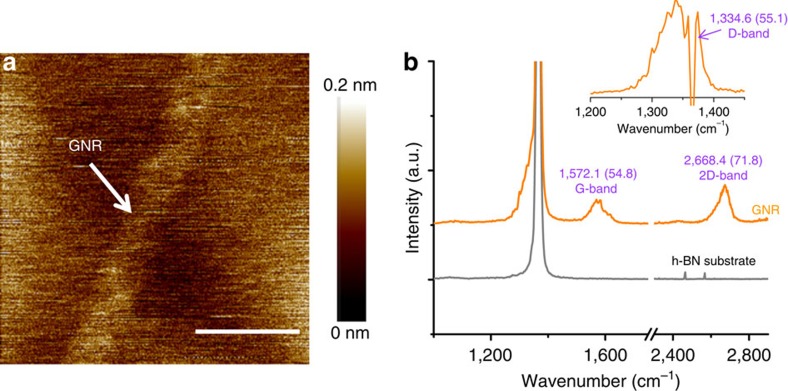
Raman spectrum of a GNR on h-BN. (**a**) AFM image of a GNR with a width of ∼15 nm. Scale bar, 30 nm; (**b**) Raman scattering of the GNR. The Raman spectrum of h-BN is also shown beside that of the GNR for comparison. The spectrum traces were normalized and shifted on the intensity axis for clarity. The inset shows the Raman spectrum of the GNR after subtracting the h-BN background. Because the position of the D-band of the GNR is very close to that of a prominent Raman peak of h-BN, such a subtraction could be utilized to identify the existence of weak Raman peaks. The full width at half-maximum (FWHM) for each peak is given in parentheses with the peak position. The band names of the main peaks are also indicated. The parameters of all Raman peaks were extracted via Lorentzian fitting, and the wavelength of the exciting laser was 488 nm.

Raman measurements were carried out to investigate the structural and electronic properties of the nanoribbons. Figure 3 shows the AFM image of a 15-nm-wide GNR and its Raman spectrum. In the spectra of Fig. 3b, a prominent sharp peak appeared at ∼1,365 cm^−1^, which was attributed to the Raman-active LO phonon of h-BN (ref. 19). For the spectrum of the GNR, the G-, D-, D′- and 2D-bands were fitted with a single-Lorentzian line shape (see Supplementary Fig. 11). The Raman spectra show a prominent G-band (∼1,572.1 cm^−1^) as well as a single-Lorentzian-shaped 2D-band (∼2,668.4 cm^−1^), which were expected for monolayer graphene. Compared to that of the pristine graphene domain^18^, the red shift of ∼9 cm^−1^ in the G-band position indicates the existence of a tensile strain of ∼0.6% in the GNR^20^,^21^. Furthermore, the GNR spectrum shows a D-band at 1,334.6 cm^−1^. At 1,617.5 cm^−1^ (D′-band), a tiny shoulder appears on the right side of the G band. Both the Raman D- and D′-bands may have primarily originated from the lattice distortions and disorder at the GNR/h-BN boundary. The 2D-band exhibits a characteristic of I(2D)/I(G)>1, also indicating the single-layer nature of the GNR, where I(2D) and I(G) represent the intensity values of the 2D and G-bands, respectively. In addition, the observed broadening of the full width at half-maximum for the Raman G- and 2D-bands may have been due to strain variations at the nanometre-scale^22^.

### Electronic transport properties of GNR transistors

**Figure 4:**
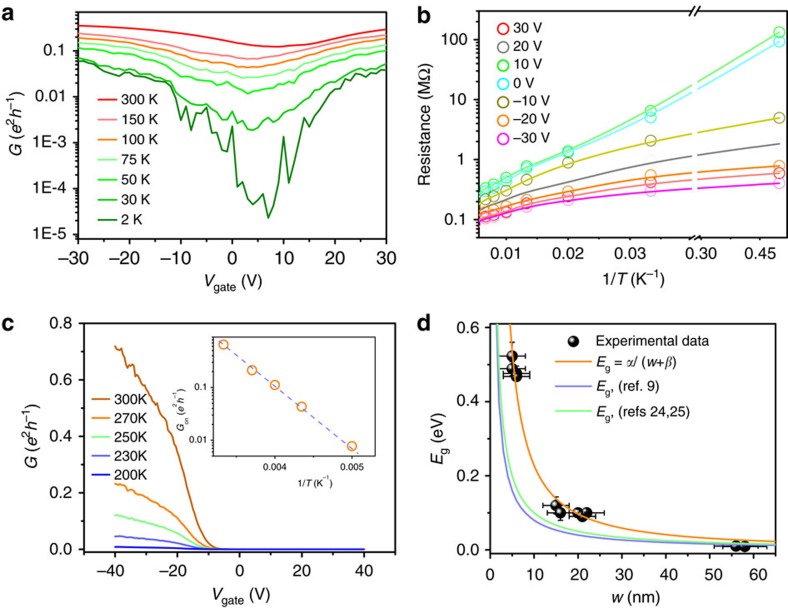
Electronic transport through GNR devices on h-BN. (**a**) Conductance (*G*) of GNRs with a width of ∼15 nm as a function of the back gate voltage (*V*_gate_) at different temperatures; (**b**) Arrhenius plot of the resistance of the 15-nm GNR FET under different *V*_gate_ values at temperatures from 2 to 250 K. The solid curves are fits based on a simple two-band (STB) model (see Supplementary Note 3); (**c**) Electronic transport through a narrow ribbon with a width of ∼5 nm. Its conductance can be completely switched off, even at 300 K. The inset shows the conductance at *V*_gate_=−30 V versus inverse temperature from 200 to 300 K. The dashed line is fit to the experimental data according to 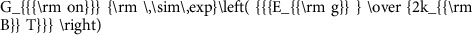; (**d**) Band gap *E*_g_ extracted from experimental data for GNRs versus their ribbon width (*w*). The orange curve is a fit of our experimental data with *E*_g_ (eV)∼*α*/(*w*+*β*) (*α* is in units of eV nm, both *β* and *w* are in units of nm). The light purple (*α*=0.8 eV nm) and light green curves (*α*=1.0 eV nm) are empirical curves adapted from ref. 9 and refs 24, 25, respectively. Error bars for the experimental data represent standard deviation of uncertainty in AFM measurement or gap extraction.

To investigate the electrical properties of the GNRs, field effect transistor (FET) devices were produced using p-type doped Si/SiO_2_ (300 nm thick for the SiO_2_ layer; for details of the device fabrication, see ‘Methods' section and Supplementary Figs 11–16). Figure 4 shows the field effect characteristics of representative GNRs at different widths. For the 15-nm GNR, the plot of conductance *G* versus *V*_gate_ at different temperatures, shown in Fig. 4a, exhibits obvious modulations due to the external electrical field. The field effect mobility extracted from the 15-nm GNR was ∼916.1 cm^2^ V^−1^ s^−1^ at 300 K. According to a simple two-band model (see Supplementary Note 3), the value of the band gap could be extracted by fitting the resistance–temperature curve (Fig. 4b; for details of the fitting, please see Supplementary Information). The fitting includes contributions from both the normal thermal activation and electrical contact resistance. The extracted band gap for the 15-nm GNR was 120±23 meV. Next, the narrowest GNRs were measured. Some devices exhibited obvious transistor behaviour, even at room temperature. As shown in Fig. 4c, a ∼5-nm GNR device had a *G*_on_/*G*_off_ ratio of >10^4^. Its conductance appeared to be completely insulating, with *G*<10^−4^ × *e*^2^ × *h*^−1^=10^−10^ S from *V*_gate_=0 to 10 V (off-state); then, it gradually switched on, exhibiting a relatively high *G*=0.7 × *e*^2^ × *h*^−1^ at room temperature for *V*_gate_=−40 V. The scattering mean free path was estimated to be ∼50 nm. The mobility of the GNR was estimated to be ∼765 cm^2^ V^−1^ s^−1^ at 300 K. Accurately measuring the off-state resistance of the narrowest GNR FET became challenging due to noise and set-up limitations. It was found that the conductance near the on-state exhibited an exponential relationship with inverse temperature from 200 to 300 K. This result indicates that a Schottky barrier dominated the conductance of the GNR FET because of the high work functions of Pd and Ni (refs 9, 23). We estimated the band gaps (*E*_g_) of the narrow GNRs by fitting the conductance near the on-state 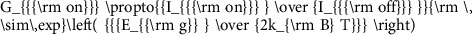, where *k*_B_ is Boltzmann's constant and *T* is temperature. The extracted band gap value for the 5-nm GNR was 489.4±19.0 meV (see the Supplementary Information for details of the fitting process). Using similar methods, the band gap values for other narrow GNRs were also estimated. As shown in Fig. 4d, the band gaps, *E*_g_, extracted from all measured GNRs were plotted with respect to the width of the corresponding GNRs. It is obvious that the band gap scaled inversely with ribbon width. The width dependence of the band gap fit well with the function *E*_g_ (eV)∼*α*/(*w+β*) (*w* is in units of nm), where parameter *α*≈1.99 eV nm and parameter *β=*−1.28 nm.

## Discussion

In the GNR samples, the band gap exhibited a strong dependence on the width of the ribbons. In particular, the sub-10-nm GNRs exhibited an electronic band gap of ∼0.5 eV. It is important to understand the possible origins of the band gap. Normally, the electronic structure of GNRs is strongly dependent on the edge of the GNRs. For this study, it was challenging to characterize the exact geometry of the narrow ribbons obtained (for example, the edge chirality and termination state of the bonds). However, the GNRs were generally parallel to the ZZ pattern of the h-BN and free of detectable edge roughness. Therefore, it is likely that most segments of the GNR edges followed a ZZ pattern. Previous studies in the literature predicted that GNRs with pristine ZZ edges would always be metallic because of their peculiar flat-band edge states localized near the Fermi level^1^. Recent theoretical papers have predicted that the flat-band would split due to electron–electron interactions, followed by the opening of a band gap caused by antiferromagnetic coupling between the spins along opposite edges of the ZZ ribbons^24^,^25^. More recently, band gaps in narrow ZZ nanoribbons have been experimentally observed in scanning tunnelling microscopy and scanning tunnelling spectroscopy studies^26^,^27^. A similar high density of states flat-band near the Fermi level was also observed in ZZ-terminated atomically sharp graphene−BN interfaces^28^. We believe that the gap opening in the GNRs embedded in h-BN may also have been due to *e*–*e* interactions. It is noted that our narrow GNRs with similar widths exhibited relatively larger band gaps than those reported in previous literature^9^,^24^. The uniaxial strain^29^ from in-plane graphene–BN bonding and the Bernal stacking^30^ on h-BN may have provided additional contributions to the gap opening in the GNRs embedded in h-BN.

The applicability of graphene for future digital devices is often questioned due to its intrinsic gapless nature. Nanoribbons offer a potential solution, but both the width and edges must be precisely controlled. In-plane graphene–BN hetero-structured films have been reported, however, only on metal surfaces^31^,^32^,^33^,^34^,^35^. In addition, control over the dimensions of graphene and graphene–BN boundaries has not been fully achieved. By employing the in-plane epitaxy of graphene in nano-trenches of h-BN, we have realized ZZ-oriented GNRs with a controlled width and smooth edges. The GNRs feature a tunable band gap, enabling sub-10-nm GNR FETs with on–off ratios >10^4^. Our results demonstrate that it is possible to resolve the fundamental gapless limitation of graphene, paving the way for the realization of graphene-based digital electronics that can operate at room temperature.

## Methods

### Etching process on h-BN surface

To prepare h-BN samples for nano-particle-assisted etching, multiple cleaning steps were adapted to ensure the reliable production of the cuts. Before depositing the h-BN flake, quartz substrates were cleaned with acetone and isopropyl alcohol and then annealed at 600 °C for 30 min to remove organic contaminants. Subsequently, the h-BN was mechanically exfoliated using semiconductor-grade tape. Next, the h-BN samples were heated in a quartz tube at 500 °C for 15 min under an Ar:H_2_ flow (850:150 sccm) to remove the tape residue. After the heat cleaning process, a solution of NiCl_2_:H_2_O at a concentration of 0.01 mg ml^−1^ was spun at 1,800 r.p.m. for 100 s onto the substrate surface and then baked for 10 min at 80 °C on a hot plate to evaporate the solvent. This NiCl_2_-treated sample was then submitted to a two-step process under an Ar:H_2_ flow (850:150 sccm): annealing at 500 °C for 20 min, which resulted in Ni nano-particle formation, and etching at 1,200 °C for 60–180 min. The optimal etching pressure was found to be ∼150 Pa.

### Growth of GNRs

Before graphene growth, etched h-BN flakes were cleaned separately in HCl solution, DI water and acetone. The substrate with the h-BN flakes was then loaded into a growth chamber. GNR growth was carried out in a low-pressure CVD furnace at 1,280 °C under an Ar flow of 10 sccm, corresponding to 15 Pa; the samples were then annealed for 5 min. Next, the Ar flow was turned off, and a C_2_H_2_ flow and a mixture of silane and Ar (5% mole ratio of silane to Ar) were introduced into the system for GNR growth. The ratio of C_2_H_2_ to silane was ∼1:1. The pressure was maintained at 5 Pa during the growth process, and the growth time was ∼5 min. After growth, both the C_2_H_2_ and silane/Ar flow were turned off, and the system was cooled to room temperature with flowing Ar. The samples of GNRs on h-BN grown on a quartz surface were moved to a highly p-type doped silicon wafer with a 300-nm-thick SiO_2_ capping layer for electrical transport studies.

### Atomic force microscopy

Cleaned samples were characterized using one AFM (Dimension Icon, Bruker), while atomic-resolution images were taken by another AFM (Multimode IV, Veeco) under ambient conditions. AFM measurements were acquired in contact mode to obtain height and friction images. SNL-10 AFM tips from Bruker, which possess a nominal tip radius of <10 nm, were used in all measurements. The use of friction contrast was necessary because this mode gives clear information about the super-structure and atomic lattice. For atomic-resolution scanning, the force constant *k* of the cantilever tips was in the range of 0.05–0.5 N m^−1^. The scan rate was set to 10–60 Hz to minimize any noise from thermal drift. The integral gain and set point were adjusted to be as low as possible during the measurement. Several hours of pre-scanning were carried out to warm up the scanner to obtain good stability during imaging. To ensure a highly accurate atomic-resolution image, scanners with a travel range of <10 μm along the *x* and *y* directions were used. Calibration at atomic resolution was performed with newly cleaved highly ordered pyrolytic graphite before measurement.

### Characterization of GNRs using Raman spectroscopy

Raman spectra were obtained with a commercially available confocal Raman instrument: model Alpha 300R from WITec. The Raman data were recorded using a laser wavelength of 488 nm 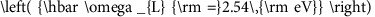. An objective lens with × 100 magnification and a 0.95 numerical aperture was used, producing a laser spot that was ∼500 nm in diameter. The laser power was maintained at <1 mW on the sample surface to avoid laser-induced heating.

### Device fabrication and transport measurements

GNR devices were created by a standard electron beam lithographic technique with Ni or Pd as the source and drain contacts on p-doped silicon wafers with 300-nm-thick SiO_2_. Next, the devices were annealed in a hydrogen atmosphere at 200 °C for 3 h to remove the resist residues and to reduce the contact resistance between the GNRs and metal electrodes before electrical measurements because the thickness of the h-BN flakes on the silicon wafer was ∼15 nm. Electrical transport measurements were carried out using a physical property measurement system (PPMS from Quantum Design, Inc.) via a Keithley 4200 semiconductor characterization system.

### Data availability

The authors declare that the main data supporting the findings of this study are available within the article and its Supplementary Information files. Additional data are available from the corresponding author upon request.

## Additional information

**How to cite this article:** Chen, L. *et al*. Oriented graphene nanoribbons embedded in hexagonal boron nitride trenches. *Nat. Commun.* **8,** 14703 doi: 10.1038/ncomms14703 (2017).

**Publisher's note**: Springer Nature remains neutral with regard to jurisdictional claims in published maps and institutional affiliations.

## Supplementary Material

Supplementary Information Supplementary Figures, Supplementary Table, Supplementary Notes and Supplementary References.
