# Supplementary material for: Oriented graphene nanoribbons embedded in hexagonal boron nitride trenches
Source: Nat Commun. 2017 Mar 9;8:14703. doi: 10.1038/ncomms14703 (PMC5347129; doi:10.1038/ncomms14703)
Supplement: Supplementary Information — Supplementary Figures, Supplementary Table, Supplementary Notes and Supplementary References. [file ncomms14703-s1.pdf]

Experiment	Atmosphere	Temperature	$NiCl_2$ Solution concentration	Etching Duration	Length(μm)	Width (nm)	Aspect ratio
#1	$Ar:H_2$=9:1, 150Pa	1200℃	0.01g/L	60min	1±0.5	~5±3nm	~200
# 2	$Ar:H_2$=9:1, 150Pa	1200℃	0.01g/L	180min	~1.8	~11±3nm	~120
# 3	$Ar:H_2$=9:1, 150Pa	1200℃	0.1g/L	60min	~1.5	~6±3nm	~260
# 4	$Ar:H_2$=9:1, 150Pa	1200℃	0.1g/L	180min	~2.5	~15±3nm	~160
# 5	$Ar:H_2$=9:1, 150Pa	1200℃	0.5g/L	10min	<0.01	N.A.	N.A.
# 6	$Ar:H_2$=9:1, 150Pa	1200℃	0.5g/L	60min	~2.2	~10nm	~220
# 7	$Ar:H_2$=9:1, 150Pa	1200℃	0.5g/L	180min	~3.8	~20±3nm	~190
# 8	$Ar:H_2$=9:1, 150Pa	1300℃	0.01g/L	60min	~1.2	~50±5nm	~25
# 9	$Ar:H_2$=9:1, 150Pa	1300℃	0.1g/L	60min	~2.3	~60±5nm	~40
# 10	$Ar:H_2$=9:1, 150Pa	1300℃	0.5g/L	60min	~3	~60±5nm	~50

Supplementary Table 1 | Summary of nano-particle assisted etching experiments under different conditions. Both the length and width of h-BN trenches exhibit some dependence on experimental parameters, such as pressure of carrying gases, temperature, the concentration of $NiCl_2$ solution, etching duration etc. The width of trenches strongly depends on temperature and duration of etching which is understandable as the etching is based on a thermal activated reaction. However, the evolution of length and the width does not seem to follow the well-known Arrhenius law, most likely due to the involvement of additional etching mechanism, for example, hydrogen-activated etching. The aspect ratios of the trenches are also calculated and insert into the table for comparison.

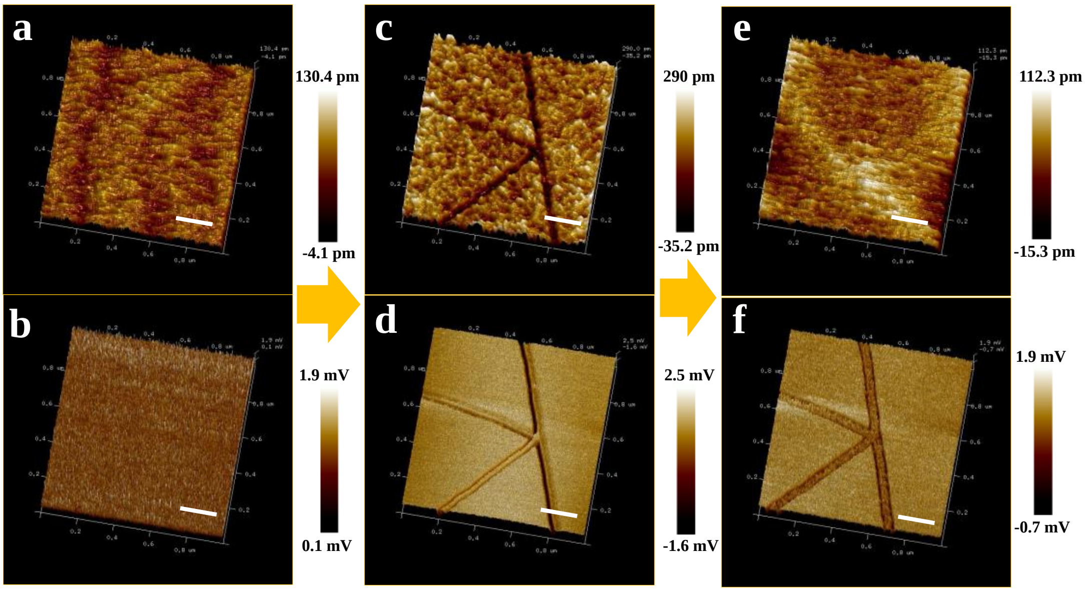

Supplementary Figure 1 | Fabrication of narrow graphene nanoribbons on the surface of h-BN. a) 3D AFM height image and b) 3D friction image of h-BN substrate taken after cleaning; c) 3D AFM height image and d) 3D friction image of ~46 nm wide h-BN nano-trenches taken after nano-particle-catalyzed-etching; e) 3D AFM height image and f) 3D friction image after the growth of graphene nanoribbons in the h-BN nano-trenches. The scale bars are 200 nm.

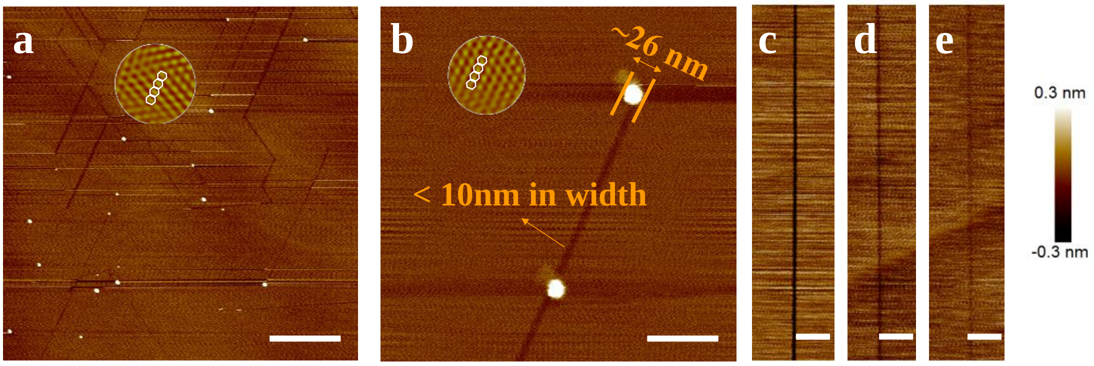

Supplementary Figure 2 | Images of nano-trenches on the surface of h-BN. (a-b) AFM height images of h-BN substrate after nano-particle-assisted-etching process, showing the existence of Ni nano-particles and sub-10 nm nano-trenches; The circular inset shows atomic-resolution friction image for h-BN, which is Fourier-filtered to improve the signal to noise ratio (SNR). The lattice structures shown in the inset confirm that the crystallographic orientations of the trenches/edges are along zigzag direction. (c-e) Images of narrow nano-trenches in a width of sub-10 nm. Scale bar in (a) is 1 μm while scale bars in (b-e) are 100 nm. The height scale for all the AFM height images is 0.6 nm.

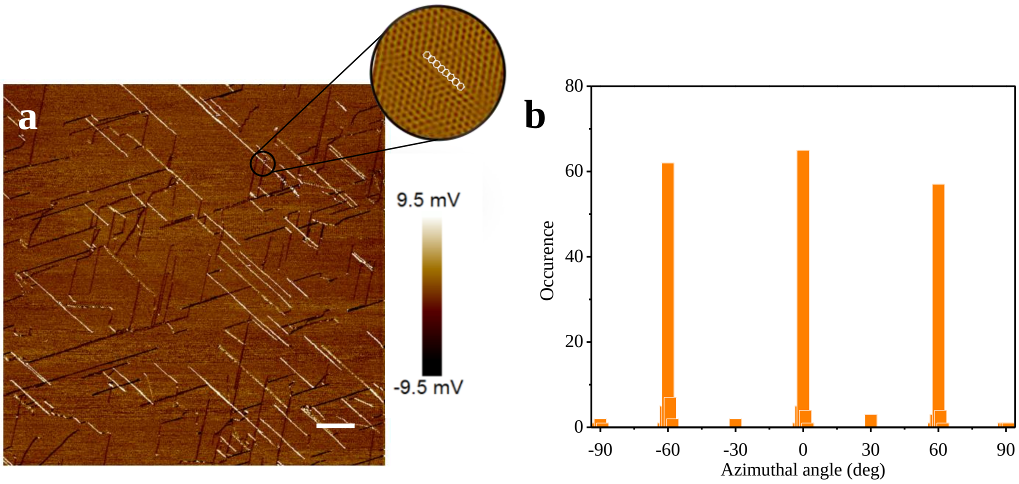

Supplementary Figure 3 | Orientation distribution of nano-trenches on h-BN. (a) Scanning probe microscope friction image of nano-trenches on h-BN. Both dark and bright lines indicate individual nano-trenches for the azimuthal-angle measurements. Scale bar: 1 μm. The circular inset shows atomic-resolution friction image for h-BN, which is Fourier-filtered to improve the signal to noise ratio (SNR). The lattice structure shown in the inset confirms that the crystallographic orientations of the trenches/edges are along zigzag direction; (b) Histogram showing the etching-direction distribution of nano-trenches on h-BN. Separation angle of 60 º relative to each other indicates an anisotropic etching along equivalent crystallographic directions.

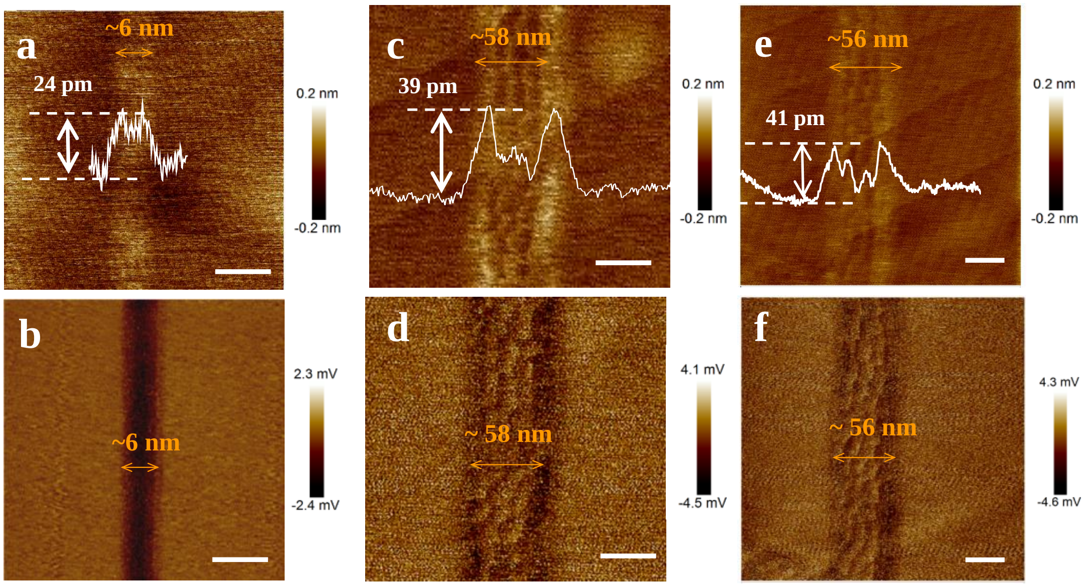

Supplementary Figure 4 | AFM Images for graphene nanoribbons on the h-BN. a) AFM height image and b) friction image of a ~ 6 nm wide graphene nanoribbon (GNR) embedded in the nano-trenches on the h-BN crystal via template growth, scale bar: 10 nm; c) AFM height image and d) friction image of a ~ 58 nm wide GNR embedded in the nano-trenches on the h-BN crystal via template growth, scale bar: 40 nm; c) AFM height image and d) friction image of a ~ 56 nm wide GNR embedded in the nano-trenches on the h-BN crystal via template growth, scale bar: 40 nm. The height profile of the line-scan across the ribbon indicates that there are obvious out-of-plane deformations along a graphene-BN in-plane boundary. The giant Moiré patterns can be observed on the wide graphene ribbon, and they are found to be stretched along the direction of GNR-h-BN boundary.

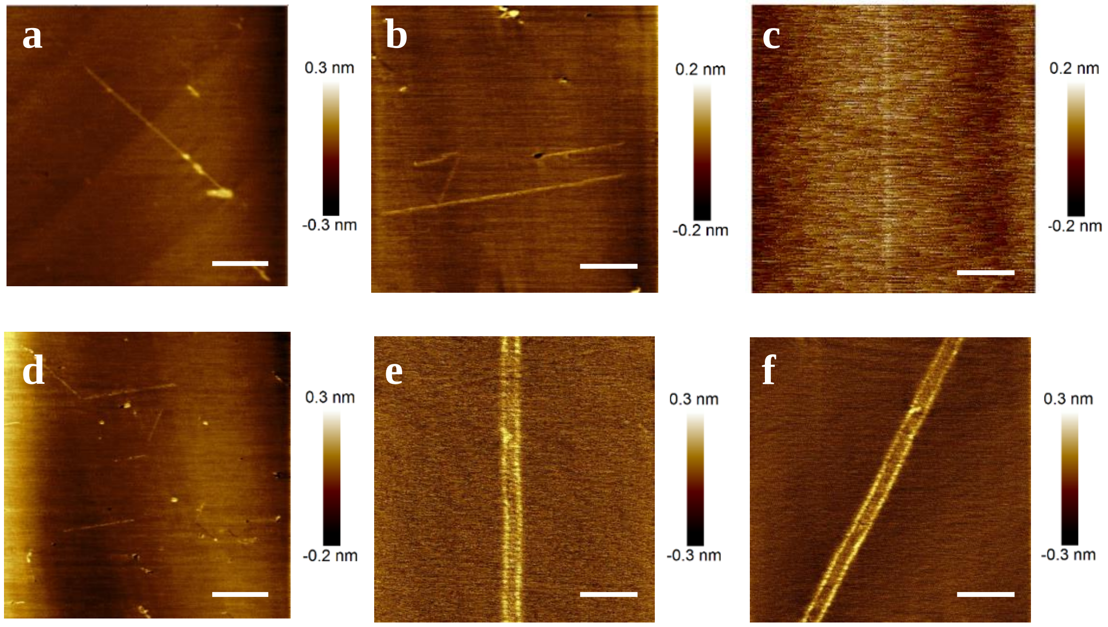

Supplementary Figure 5 | Images of graphene nanoribbons embedded in h-BN nano-trench. (a-d) AFM height images of h-BN substrate after graphene nanoribbon (GNR) growth process, showing the existence of sub-10 nm GNRs; (e-f) AFM height images of about 60 nm-wide GNR on h-BN substrate. The length of GNRs is always more than 1 μm. The scale bars in (a), (b), and (d) are 400 nm, 300 nm, and 600 nm, respectively; and the scale bars are 200 nm in (c), (e) and (f).

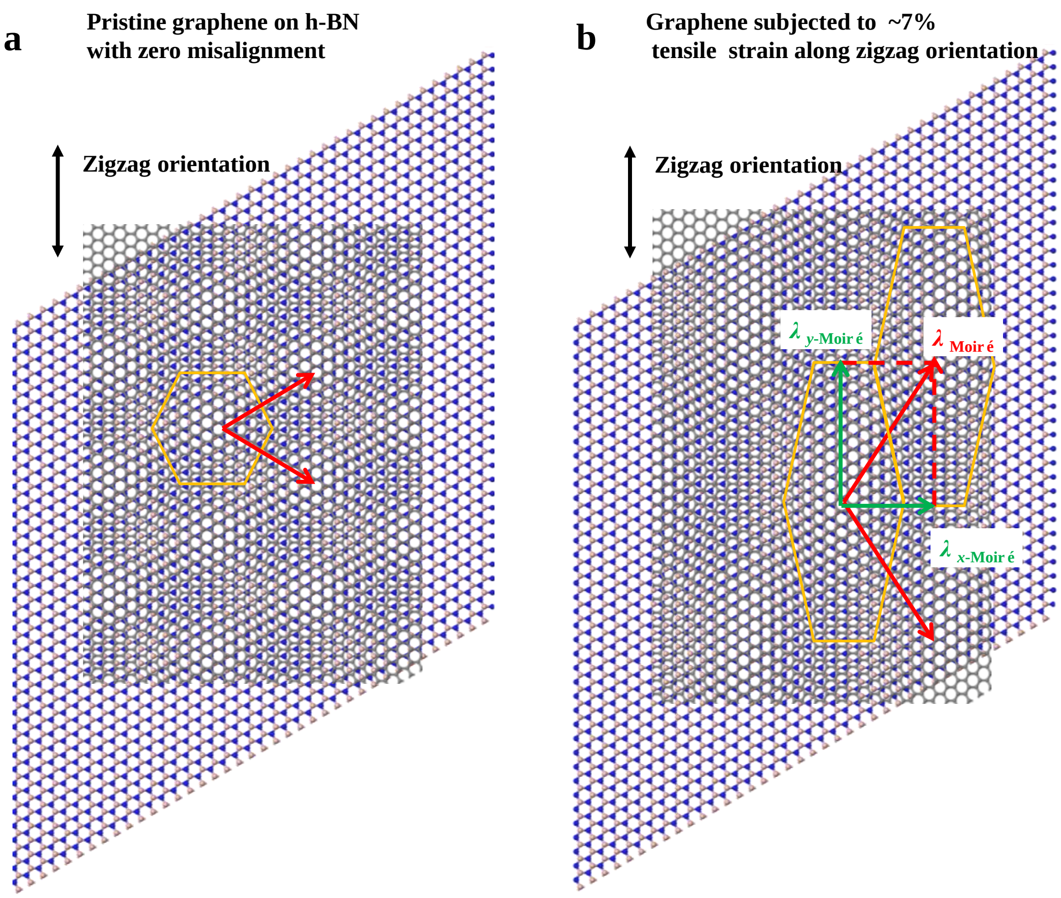

Supplementary Figure 6 | Illustration of stretched giant Moiré pattern caused by strain in graphene. (a) Sketch of graphene on h-BN for zero misalignment and an exaggerated lattice mismatch of ~10%. The two superimposed hexagonal lattices cause the emergence of a Moiré pattern. (b) The Moiré pattern is stretched by the tensile strain in graphene along zigzag orientation. $\lambda_{\text{Moiré}}$ represents the lattice vector for stretched Moiré pattern. $\lambda_{y\text{-Moiré}}$ and $\lambda_{x\text{-Moiré}}$ represents the projection of $\lambda_{\text{Moiré}}$ along the zigzag and armchair orientation, respectively.

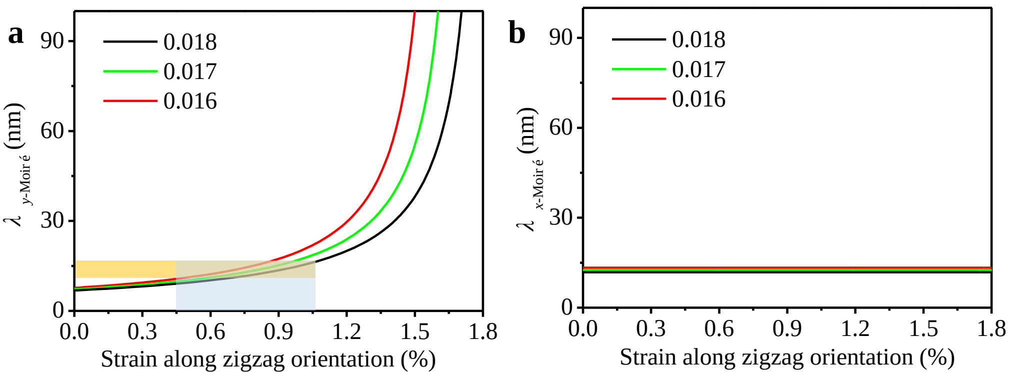

Supplementary Figure 7 | The wavelength dependence of stretched giant Moiré pattern on strain in graphene precisely aligned with h-BN. (a) $\lambda_{y\text{-Moiré}}$ versus strain in graphene along zigzag direction; (b) $\lambda_{x\text{-Moiré}}$ versus strain in graphene along zigzag direction. Here, cases of the lattice mismatch between graphene and h-BN from 0.016 to 0.018 are simulated.

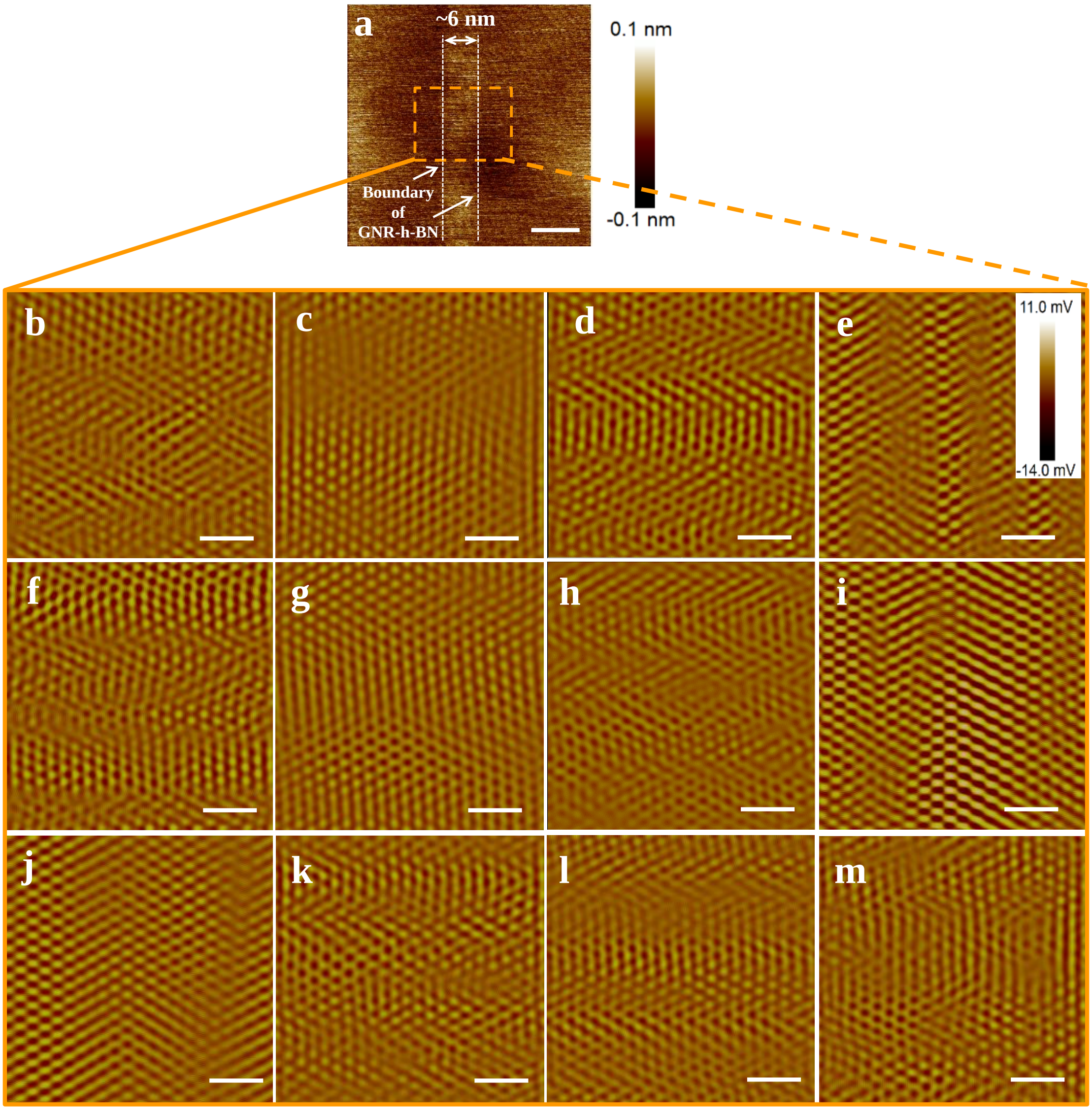

Supplementary Figure 8 | Atomic-resolution-AFM investigation on graphene epi-ribbon embedded in h-BN nano-trench. (a) AFM height image of graphene nanoribbon (GNR) embedded in h-BN, where the width of GNR is about 6 nm; (b-m) Atomic resolution scans (5×5 nm^2) taken spatial-continuously from the orange box (20×15 nm^2) in (a). These atomic images confirm that GNR and the h-BN top-layer form into a uniform monolayer during the CVD growth. By careful examination of atomic resolution AFM, no obvious boundary was found. These AFM images clearly show adjacent GNRs grown from opposite step-edge of top h-BN layer have same lattice orientation and coalesce without grain boundary defects. It is also confirmed that the GNR and adjacent h-BN layer have the same lattice orientation and merges at the boundary seamlessly. The scale bars are 10 nm in (a) and 1 nm in (b-m).

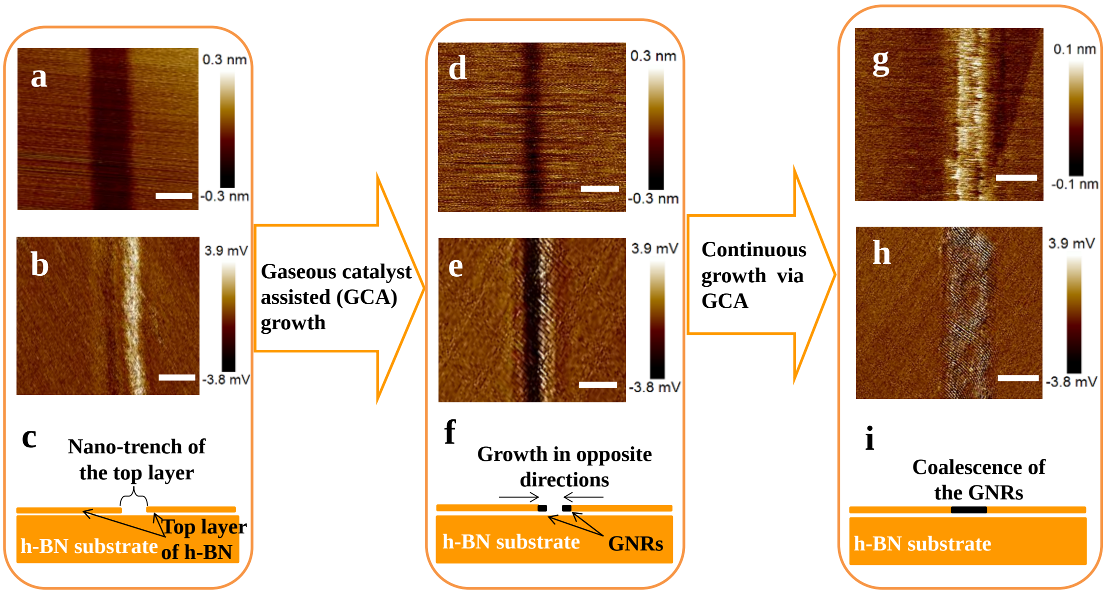

Supplementary Figure 9 | Formation stages of graphene nanoribbon embedded in h-BN nano-trench. AFM height images, friction images and schematics, for a graphene epi-ribbon formed in a nano-trench of h-BN top-layer at different formation stages: (a), (b) and (c) after the formation of h-BN nano-trenches; (d), (e) and (f) after the beginning of graphene nanoribbons (GNRs) growth from opposite sides of the nano-trench; (g), (h) and (i) after GNRs grown from opposite sides merge together. The scale bars are 20 nm.

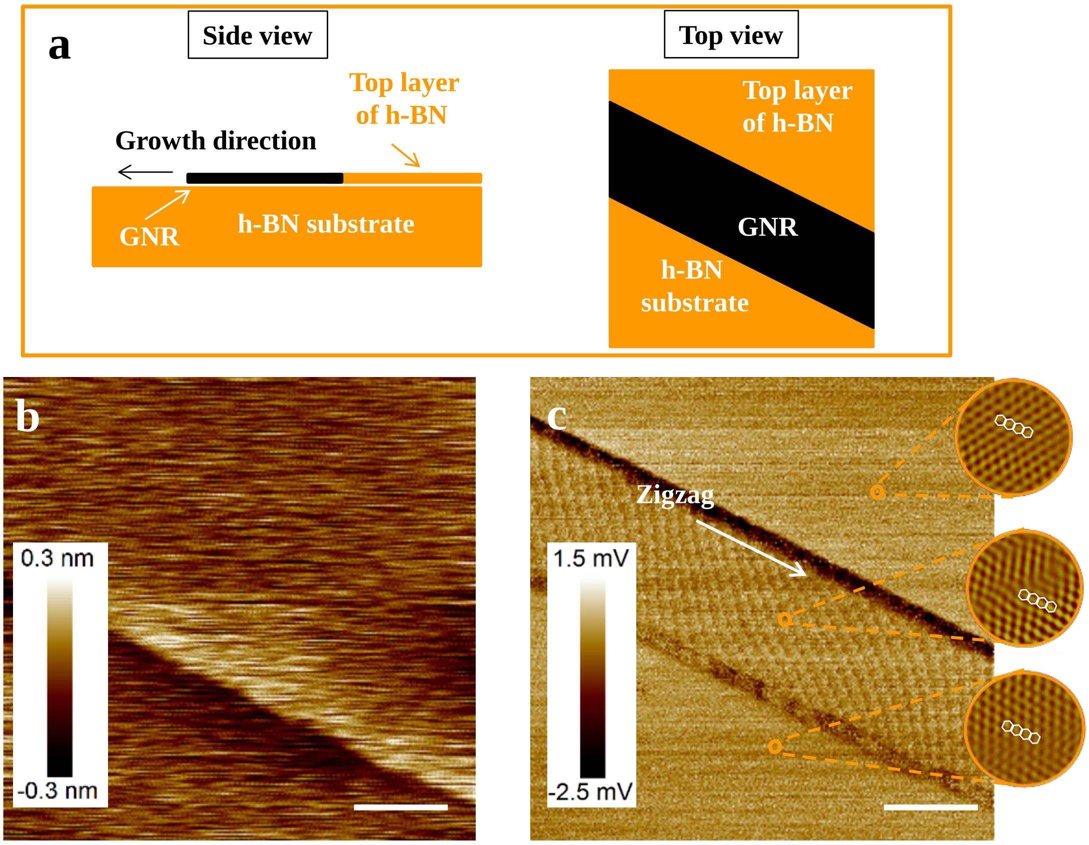

Supplementary Figure 10 | Graphene epi-ribbon along a h-BN step-edge. (a) Schematics for a graphene epi-ribbon formed along a step-edge of h-BN top-layer. Height (b) and friction (c) AFM image of the graphene nanoribbon (GNR). The height image reveals that the graphene ribbon seamlessly connects to the h-BN step-edge. In the friction image, the giant Moiré patterns with the periodicity of about 14nm can be clearly seen on the wide GNR. The circular insets in (c) show atomic-resolution friction images (from top to bottom) for the template h-BN top-layer, graphene epi-ribbon and the substrate h-BN layer, respectively. The GNR and h-BN top-layer are found to have the same lattice orientation and merges at the boundary seamlessly. The scale bars in (b) and (c) are 100 nm.

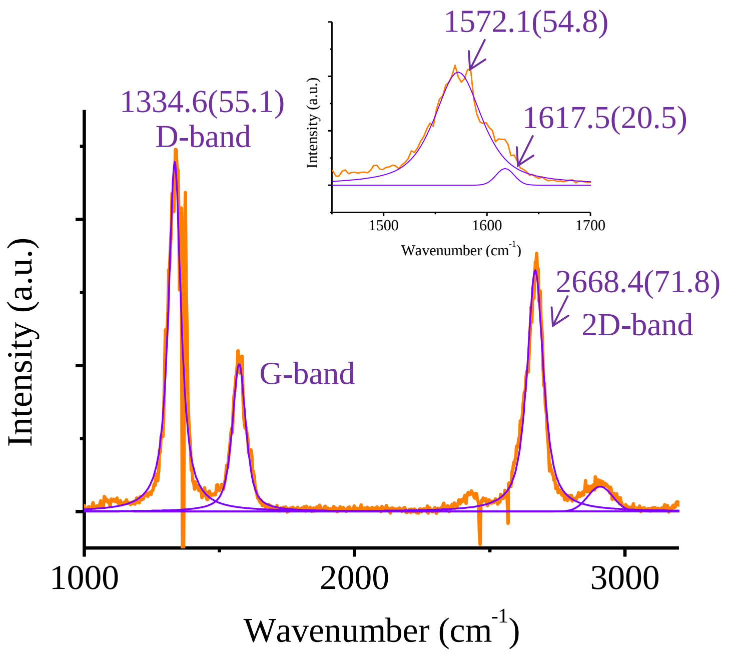

Supplementary Figure 11 | Raman spectrum of a 15 nm-wide graphene nanoribbon after subtracting the h-BN background signal. Raman spectrum of h-BN is taken beside the GNR for comparison. All spectrum-traces are normalized by using the prominent sharp peak of h-BN substrates as an intensity reference. Raman spectrum of the GNR can be obtained via subtracting h-BN backgrounds. The G-, D- and 2D- bands were fitted with a single Lorentzian line shape. Inset shows enlarged G-band peak of the GNR. A tiny shoulder (D'-band) appears on the right side of G-band. The full-width at half-maximum (FWHM) for each peak is given in parentheses with the peak location value. The wavelength of the exciting laser is 488 nm, the laser power density incident on the sample surface is less than 1 mW over a 1 μm spot diameter, and the integration time took around 120 s for each spectrum.

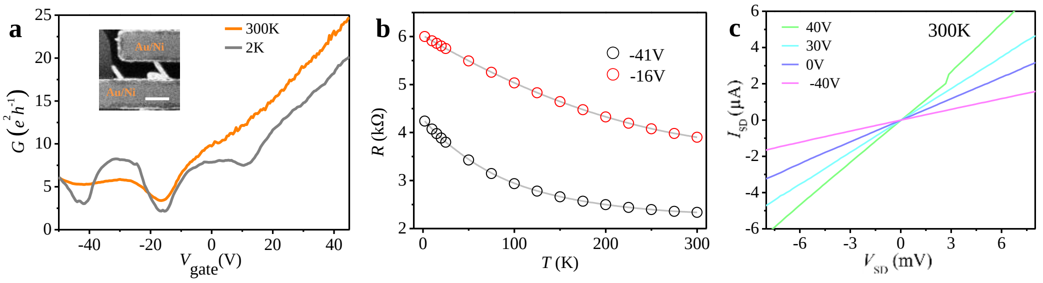

Supplementary Figure 12 | Electronic transport through a ~58 nm graphene nanoribbon device on h-BN. (a) Conductance (G) of a graphene nanoribbon (GNR) with a width of ~58 nm as a function of back gate voltage (V_{gate}). In the 58 nm-wide GNR, the conductance exhibits three dips at the Dirac point and Secondary Dirac point (SDP), which indicates the existence of giant super-lattice. Inset shows a scanning electron micrograph (SEM) image of the nanoribbon channel corresponding to the transfer curves. The scale bar is 1 μm. The channel length is about 1 μm. Its field effect mobility is about ~3 053 $cm^2V^{-1}s^{-1}$ at 300 K; (b) Resistance (R) under different V_{gate} versus temperature (T) from 2 to 300 K for the 58 nm-wide GNR, the solid curves are fits based on a simple two-band model, the extracted band gap is about 9 ±2 meV; (c) I_{SD}-V_{SD} curves at gate biases V_{gate} ranging from -40 V to 40 V at room temperature.

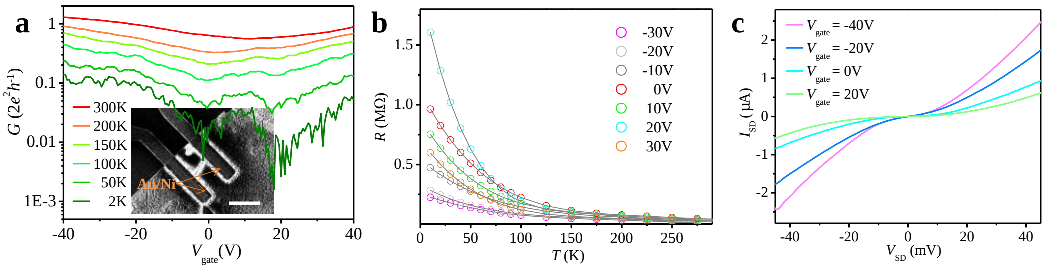

Supplementary Figure 13 | Electronic transport through a ~20 nm graphene nanoribbon device on h-BN. (a) Conductance (G) of a graphene nanoribbon (GNR) with a width of ~20 nm as a function of back gate voltage (V_{gate}). Inset shows SEM image of the nanoribbon channel corresponding to the transfer curves. The scale bar is 2 μm. The channel length is about 1.3 μm; Its field effect mobility is about 1 033 $cm^2V^{-1}s^{-1}$ at 300 K; (b) Resistance (R) under different V_{gate} versus temperature (T) from 10 to 300 K for the 20 nm-wide GNR, the solid curves are fits based on a simple two-band model, the extracted band gap is about 100 ±11 meV; (c) I_{SD}-V_{SD} curves for the device in (a) at different gate biases at room temperature.

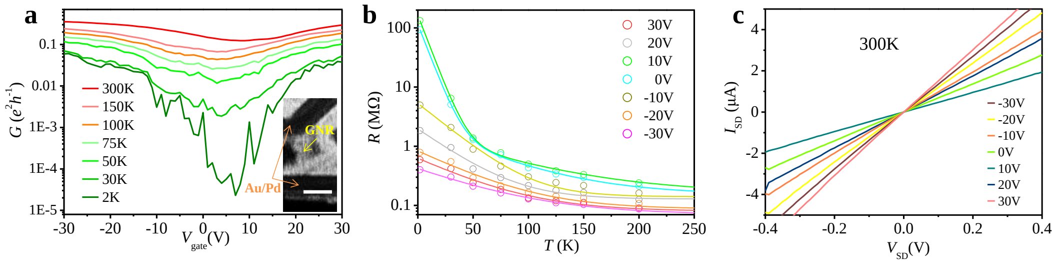

Supplementary Figure 14 | Electronic transport through a ~15 nm graphene nanoribbon device on h-BN. (a) Conductance (G) as a function of back gate voltage (V_{gate}). Inset shows SEM image of the graphene nanoribbon (GNR) channel corresponding to the transfer curves. Scale bar in the inset is 1 μm. The channel length of FET is about 1.5 μm, its field effect mobility is about 916.1 cm^{2}V^{-1}s^{-1} at 300 K; (b) Resistance (R) under different V_{gate} versus temperature (T) from 25 to 300 K for the 20 nm-wide GNR, the solid curves are fits based on a simple two-band model, the extracted band gap is about 120±23 meV; (c) I_{SD}-V_{SD} curves for the device in (a) at different gate biases at room temperature.

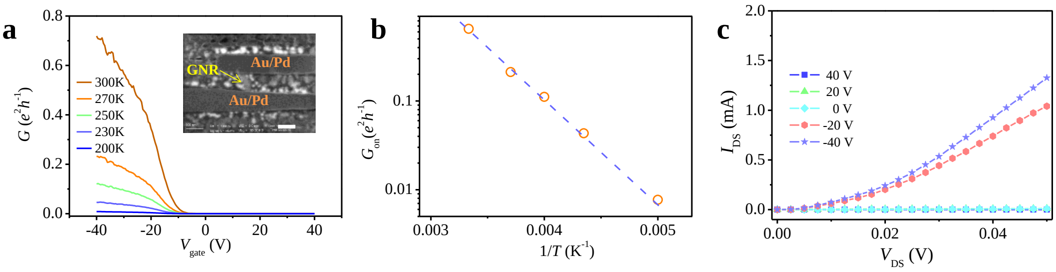

Supplementary Figure 15 | Electronic transport through a ~5 nm graphene nanoribbon device on h-BN. Conductance (G) of another graphene nanoribbon (GNR) with an estimated width of about 5 nm. Its conductance can be completely switched-off even at 300 K. Inset shows SEM of the nanoribbon channel corresponding to the transfer curves. The channel length is about 300 nm. Scale bar in the inset is 300 nm. Its field effect mobility is about 765.8 $cm^2V^{-1}s^{-1}$ at 300 K. The GNR field effect transistor (FET) in a channel length of about 300 nm exhibits rather high G=0.7 e^2h^{-1} at room temperature when V_{gate}= -40 V. Charge carrier was scattered for (4 e^2h^{-1})/(0.7 e^2h^{-1})≈5 times. The scattering mean free path (MFP) is estimated to be about 300 nm/(5+1)=50 nm even without excluding the contribution from the contact; (b) Inset shows the relationship of conductance at V_{gate}=-30 V versus inverse temperature (T) from 200 to 300 K, the dashed line is fit to the experimental data according to $G_{on}\sim\exp(\frac{E_g}{2k_BT})$. The extracted band gap is about 489±19 meV; (c) I_{SD}-V_{SD} curves for the device in (a) at gate biases V_{gate} ranging from -40 V to +40 V.

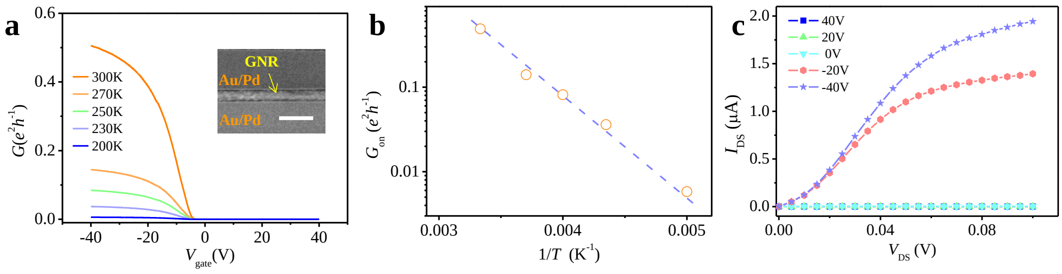

Supplementary Figure 16 | Electronic transport through a ~5 nm graphene nanoribbon device on h-BN. Conductance (G) of another graphene nanoribbon (GNR) with an estimated width of about 5 nm. Its conductance can be completely switched-off even at 300 K. Inset shows SEM of the nanoribbon channel corresponding to the transfer curves. The channel length is about 500 nm. Scale bar in the inset is 1 μm. Its field effect mobility is about 790.8 $cm^2V^{-1}s^{-1}$ at 300 K; The GNR FET in a channel length of about 500nm exhibits rather high G=0.5 e^2h^{-1} at room temperature when V_{gate}= -40 V. Charge carrier was scattered for (4 e^2h^{-1})/(0.5 e^2h^{-1})≈8 times. The scattering mean free path (MFP) is estimated to be about 500 nm/(8+1)≈55.6 nm; (b) Inset shows the relationship of conductance at V_{gate}=-30 V versus inverse temperature (1/T) from 200 to 300 K, the dashed line is fit to the experimental data according to $G_{on}\sim\exp(\frac{E_g}{2k_BT})$. The extracted band gap is about 523±37.2 meV; (c) I_{SD}-V_{SD} curves for the device in (a) at gate biases V_{gate} ranging from -40 V to +40 V.

Supplementary Note 1: Simulation of Stretched Moiré Pattern

The schematic in Supplementary Fig. 6 is an explanation to Moiré geometry. When h-BN is overlaid with a graphene layer, new symmetry may emerge leading to the Moiré pattern. Its orientation and wavelength are fixed and can be derived mathematically. The hexagonal lattice of h-BN is defined by vectors (a_1, a_2), where a_1 and a_2 can be written as $a_1=a(\frac{\sqrt{3}}{2}, -\frac{1}{2})$ and $a_2=a(\frac{\sqrt{3}}{2}, \frac{1}{2})$, respectively. a represents the lattice constant of h-BN. Similarly, graphene lattice is represented by (b_1, b_2). The lattice of precisely-aligned graphene on h-BN substrate is shorter in length by a lattice mismatch factor of δ in the range 1.6% to 1.8%. The factor δ denotes lattice mismatch between h-BN and graphene. b_1 and b_2 can be written as $b_1=a(1\text{-}\delta)(\frac{\sqrt{3}}{2}, -\frac{1}{2})$ and $b_2=a(1\text{-}\delta)(\frac{\sqrt{3}}{2}, \frac{1}{2})$, respectively. If strain (β) is applied to graphene along zigzag orientation, the b_1 and b_2 can be written as $b_1=a(1\text{-}\delta)(\frac{\sqrt{3}}{2}(1+\beta), -\frac{1}{2})$ and $b_2=a(1\text{-}\delta)(\frac{\sqrt{3}}{2}(1+\beta), \frac{1}{2})$, respectively. The reciprocal lattice vectors of the Moiré pattern is given by: $k_{\text{Moiré}}=k_{\text{graphene}}-k_{\text{h-BN}}$ Therefore, the Moiré pattern in real space can be directly derived by numerical method. Here we use 2.50 Å as the value of a. The projection ($\lambda_{y\text{-Moiré}}$ and $\lambda_{x\text{-Moiré}}$) for the wavelength of Moiré pattern ($\lambda_{\text{Moiré}}$) as functions of tensile strain (β from 0~1.8%) in graphene along zigzag direction are plotted in Supplementary Fig. 7 with lattice mismatch (δ) in the range 1.6% to 1.8%. Here, $\lambda_{y\text{-Moiré}}$ and $\lambda_{x\text{-Moiré}}$ represents the projection of $\lambda_{\text{Moiré}}$ along the zigzag and armchair orientation, respectively.

It is found that the value of $\lambda_{y\text{-Moiré}}$ increases with the increase of strain (β) while the value of $\lambda_{y\text{-Moiré}}$ keeps untouched. We noticed that $\lambda_{y\text{-Moiré}}$ has a measured value of 13~16 nm in stretched Moiré patterns. (See Fig. 2 and Supplementary Fig. 4). According to the plot in Supplementary Fig. 7, The $\lambda_{y\text{-Moiré}}$ ranging from 13 to 16 nm yields estimated β of 0.45~1.05%. In other words, the GNR aligned with h-BN substrate suffers corresponding tensile strain of 0.75% ±0.3%.

Supplementary Note 2: Capacitance and Field Effect Mobility

Electronic transport measurements in two-terminal configuration were also carried out to characterize the GNRs grown on h-BN. The gate voltage (V_g) dependence of the conductance at different temperature is plotted in Fig. 4 and Supplementary Fig. 12-16. The carrier mobility and on-off ratio of each GNR device can be extracted from the plots.

In a classical regime, the capacitance is completely determined by the object's geometry and a dielectric constant of the medium. If the object's size shrinks to a nanometer scale, a finite DOS which originates from the Pauli exclusion principle should be considered. Low-dimensional systems, having a small DOS, are not able to accumulate enough charge to completely screen the external field. In order to describe the effect of the electric field penetration in a finiteDOS system, quantum capacitance should be taken into account. According to the theoretical calculation (Phy. Rev. B 80, 205402 (2009)), the quantum capacitance can be ignored in devices with thick dielectric layer. As we are using 300 nm SiO_2 as dielectric layer in these experiments, only classical capacitance is taken into account.

We used three-dimensional electrostatic simulation to calculate effective capacitance (C_{gs}). Fast Field Solvers Software is available at http://www.fastfieldsolvers.com. The simulated structure includes a large highly-doped-Si plane as back-gate, a dielectric bi-layer with the same lateral dimension as back-gate (300 nm thickness and dielectric constant ε_0=3.9, for the bottom SiO_2 layer and 10 nm thickness and ε_0=4 for the h-BN top-layer) and a graphene layer with experimental dimension lying ~0.35 nm above the dielectric layer and two metal fingers with experimental dimension to represent contacts. ~1 nm grids for GNR in a width of *w* (nm) are used in simulation. And then, the capacitances are obtained: C_{gs} = 45.1 pF·m^{-1} for a *w*=58 nm ribbon (Supplementary Fig. 12), C_{gs} = 5.9 pF·m^{-1} for a *w*=20 nm ribbon (Supplementary Fig. 13), C_{gs} = 5.2 pF·m^{-1} for a *w*=15 nm ribbon (Supplementary Fig. 14) and C_{gs} = 3.98 pF·m^{-1} for a *w*=6 nm ribbon; C_{gs} = 3.71 pF·m^{-1} for a *w*=5 nm (Supplementary Fig. 15 and 16). Based on standard transistor model, the intrinsic carrier mobility is $\mu=\frac{\frac{\mathrm{d}G}{\mathrm{d}V_{gs}}\cdot L}{C_{gs}}$, where L represents the channel length of

GNRs; G represents the channel conductance measured. And then the electrical field mobilities can be derived for the GNR transistors at 300 K (shown in supplementary Fig. 12-16).

Supplementary Note 3: Simple Two Band (STB) Model

For graphitic materials,[1-4] a simple two band (STB) model are always used to extract the band gap information from the temperature-dependence of resistance measured. Based on this model, the densities of electrons (n) and holes (p) are given by $n=C_n k_B T \ln(1+\exp(-\frac{E_C-E_F}{k_B T}))$ and $p=C_p k_B T \ln(1+\exp(-\frac{E_F-E_V}{k_B T}))$, respectively. Here, E_F, E_C, E_V are the energies at the Fermi level, bottom of conduction band and top of valance band, respectively, k_B is the Boltzmann constant and C_n, C_p are constants independent of temperature (T). Ignoring the contribution from static scattering centers, the mobility of carriers can be expressed as $\mu_e=A_1 T^{-1}$ or $\mu_h=A_2 T^{-1}$, where A_1 and A_2 are constants depending on the strength of electron and hole-phonon scattering in graphite. Since the resistivity is given by $\rho=(n\mu_e e+p\mu_h e)^{-1}$, the temperature dependence of resistance can be expressed as:

$$R=\frac{P_1}{\ln(1+\exp(-\frac{E_C-E_F}{k_B T}))+P_2 \ln(1+\exp(-\frac{E_F-E_V}{k_B T}))}+R_{contact}$$

Using E_C-E_F, E_F-E_V, P_1, P_2 and $R_{contact}$ as the fitting parameters, this model fits well with our experimental data for the most samples at different V_{gate}, as shown in supplementary Fig. 12b, 13b and 14b. Based on this model, the energy gaps obtained for the three samples are 9±2 meV, 100±11 meV and 120±23 meV for the GNR samples with a width of 58 nm, 20 nm and 15 nm, respectively. The model can rule out the contribution from the contacts (e.g. Schottky barrier).

With the decrease of GNR width, the GNR resistance greatly increases. In addition, the current measured decreases greatly and becomes very sensitive to influence from the environment. As such, resistance of GNR cannot be determined accurately. On the other hand, the influence from Schottky barriers becomes pronounced. Therefore, the STB model cannot be applied to the sub-10 nm GNR with relatively large E_g.

Supplementary Note 4: Band Gap Estimation in Ultra-narrow GNRs

For the ultra-narrow GNR, it is found that the conductance at on-state exhibits an exponential relationship with inverse temperature from 200 K to 300 K. It indicates that Schottky barriers (SB) dominate the conductance of the GNR FET because of high work function of Pd and Ni [5,6]. We would like to estimate the band gap (E_g) of the narrow GNRs by fitting the conductance near on-state $G_{on} \propto \frac{I_{on}}{I_{off}} \sim \exp(\frac{\varnothing_{SB}}{k_B T})$ (where k_B is the Boltzmann's constant, T is temperature and $\varnothing_{SB}$ is the height of the Schottky barrier). Here $\varnothing_{SB}$ is about half of the gap E_g. Thus, $G_{on} \sim \exp(\frac{E_g}{2k_B T})$ [7-9] The extracted values of band gaps for the 5-6 nm GNRs are 489.4±19.0 meV, 523±37.2 meV, 468±7.2 meV and 476±20 meV, respectively.

Supplementary References

1 Novoselov, K.S. *et al*., Electric field effect in atomically thin carbon films. *Science* **306**, 666-669 (2004).

2 E. Dujardin, *et al*., Fabrication of mesoscopic devices from graphite microdisks. *Appl. Phys. Lett.* **79**, 2474-2476 (2001).

3 Wang, H., Choong, C., Zhang, J., Teo, K. L. and Wu, Y. Differential conductance fluctuation of curved nanographite sheets in the mesoscopic regime. *Solid State Communications* **145**, 341-345 (2008).

4 C. Berger, *et al*., Electronic confinement and coherence in patterned epitaxial graphene. *Science* **312**, 1191-1196 (2006).

5 Javey, A., Guo, J., Wang, Q., Lundstrom, M. & Dai, H. Ballistic carbon nanotube field-effect transistors. *Nature* **424**, 654-657 (2003).

6 Li, X., Wang, X., Zhang, L., Lee, S. & Dai, H. Chemically derived, ultrasmooth graphene nanoribbon semiconductors. *Science* **319**, 1229-1232 (2008).

7 Chen, Z., Lin, Y.-M., Rooks, M. J., Avouris, P. *Physica E: Low-dimensional Systems and Nanostructures*, **40**, 228-232 (2007).

8 Xia, F., Farmer, D., Lin, Y., and Avouris, P. Graphene field-effect-transistors with high on/off current ratio and large transport band gap at room temperature. *Nano Letters* **10**, 715-718 (2010).

9 Hwang, W. S. *et al*., Graphene nanoribbon field-effect transistors on wafer-scale epitaxial graphene on SiC substrates. *APL Mater.* **3**, 011101 (2015).
